# The Effects of the Processing of Positive Memories Technique on Posttrauma Affect and Cognitions Among Survivors of Trauma: Protocol for a Daily Diary Study

**DOI:** 10.2196/51838

**Published:** 2024-01-12

**Authors:** Talya Greene, Ateka A Contractor, Sheila Daniela Dicker-Oren, Andrea Fentem, Sharon R Sznitman

**Affiliations:** 1 Department of Clinical, Educational and Health Psychology University College London London United Kingdom; 2 Department of Community Mental Health University of Haifa Haifa Israel; 3 Department of Psychology University of North Texas Denton, TX United States; 4 School of Public Health University of Haifa Haifa Israel

**Keywords:** affect, case series design, cognitions, experience sampling, intensive longitudinal assessment, positive autobiographical memories, posttrauma health, posttraumatic stress disorder, trauma survivors

## Abstract

**Background:**

The Processing of Positive Memories Technique (PPMT) is a promising new treatment approach for posttraumatic stress disorder (PTSD), which involves detailed narration and processing of specific positive autobiographical memories. Indeed, preliminary case-series studies have found reductions in PTSD symptoms, negative affect, and negative cognitions among survivors of trauma who have received PPMT. However, PPMT’s effects have not been investigated at the daily level. In this study, we describe the protocol for a study that will examine the daily-level impacts of PPMT in a trauma-exposed, nonclinical community sample.

**Objective:**

This study uses an innovative research protocol that combines case-series design and daily diary approaches to examine changes in daily affect, daily cognitions, and daily PTSD symptoms pre- and post-PPMT. We hypothesize that at the daily level, in comparison to their own pre-PPMT levels, following the PPMT intervention, participants will report (1) a lower count of endorsed daily PTSD symptoms, (2) increases in daily positive affect and decreases in daily negative affect, (3) increases in positive affect reactivity to daily positive events, and (4) decreases in daily posttrauma cognitions.

**Methods:**

We are currently recruiting participants (target n=70) from a metroplex in the southwest United States. Following a screening survey, eligible participants complete a preintervention baseline survey, followed by 21 daily surveys in their natural environments. Then, they receive 4 PPMT sessions on a weekly basis. After the conclusion of the PPMT intervention, participants complete a postintervention outcome survey and 21 daily surveys. To compare daily affect, daily cognitions, and daily PTSD symptoms before and after PPMT, we will use the daily diary report data and conduct multilevel random intercepts and slopes linear regression models.

**Results:**

Data collection was initiated in March 2022 and is expected to end by June 2024. As of November 28, 2023, a total of 515 participants had consented to the study in the screening phase. No analyses will be conducted until data collection has been completed.

**Conclusions:**

Study findings could clarify whether deficits in positive autobiographical memory processes may also characterize PTSD alongside deficits in traumatic memory processes. Furthermore, PPMT could be an additional therapeutic tool for clinicians to help clients reduce posttraumatic distress in their everyday lives.

**International Registered Report Identifier (IRRID):**

DERR1-10.2196/51838

## Introduction

Posttraumatic stress disorder (PTSD) is a psychological condition that can develop following exposure to traumatic events, characterized by intrusive thoughts and re-experiencing symptoms, avoidance of trauma reminders, negative cognitions and mood, and heightened arousal [1]. A proposed mechanism underlying the development and maintenance of PTSD is disruptions in the encoding, consolidation, and retrieval of negatively- and positively-valenced autobiographical memories [2-4]. Evidence suggests that survivors of trauma with PTSD report difficulties accessing and detailing positive autobiographical memories [5-7], akin to reported difficulties with traumatic autobiographical memories [8]. Unsurprisingly, PTSD interventions typically target the content, processes, and phenomenological characteristics of autobiographical memories [9,10]. While such trauma-focused interventions (eg, prolonged exposure and cognitive processing therapy) are effective for many survivors of trauma, a significant proportion of survivors of trauma do not respond to these treatments, and there is a high degree of dropout from these interventions [11]. This highlights the need to develop alternative therapeutic approaches.

While most PTSD interventions address engagement with only traumatic autobiographical memories, only a few other interventions address both traumatic and positive autobiographical memories or only positive autobiographical memories and have been shown to be effective. For instance, Memory Specificity Training (targeting one’s ability to retrieve specific autobiographical memories irrespective of valence) is effective for PTSD and posttrauma distress [12,13]. Also, Broad-Minded Affective Coping, a positive emotion induction technique through the retrieval of positive autobiographical memories, improves mood among individuals with PTSD [14]. Such evidence suggests that a focus on positive autobiographical memories may be a helpful target in PTSD interventions.

The PTSD-Positive Memory Model [15,16] outlines that when survivors of trauma repeatedly retrieve, relive, and detail specific positive autobiographical memories, they may experience an improvement in PTSD symptoms, affect, and beliefs over time [15]. This model was foundational to the development of the Processing of Positive Memories Technique (PPMT), which is a 4- to 5-session intervention tailored to PTSD symptoms. During PPMT sessions, survivors of trauma are guided to narrate details of salient positive autobiographical memories; to access and strengthen positive values, affect, strengths, and thoughts associated with these memories; and to engage in positive affective, cognitive, and behavioral changes [17]. PPMT is influenced by positive psychology, a field that emphasizes factors and mechanisms that enhance psychological well-being rather than focusing solely on pathology [18,19]. PPMT draws from positive psychology interventions (eg, sharing positive narratives with others and using mental imagery to re-expereince positive events) [19-21] and from interventions that increase memory retrieval to improve mental health [22]. The detailed session-by-session content of PPMT is outlined by Contractor and colleagues [17].

Practicing PPMT may help survivors of trauma retrieve more positive autobiographical memories over time, which may also translate to retrieving fewer negative autobiographical memories. Consequently, survivors of trauma may be able to better contextualize and integrate traumatic autobiographical memories with existing beliefs [23] and with other memories [2,24], which in turn could aid recovery after a trauma [2,25]. Positive autobiographical memories may also become primary reference points to interpret experiences and influence self-concept [26-28]. Furthermore, by repeatedly retrieving positive autobiographical memories and associated content, survivors of trauma may lessen their focus on negative material, experience more positive affect, and downregulate negative affect [29-32]. This may be especially helpful for survivors of trauma who experience emotional distress from retrieving negative autobiographical memories. In turn, this improved affect may help survivors of trauma positively interpret events [29,33] and note more positive content in their thoughts [34]. Overall, retrieving positive autobiographical memories may improve well-being [35], resilience [36], and adaptive coping [37], serving as a reminder that there are positive values and thoughts to hold on to despite the hardships faced by survivors of trauma.

Pilot studies have shown that PPMT is feasible and may improve therapeutic outcomes for survivors of trauma. Using an experimental design, a 2-session modified-PPMT [38] and a 5-session PPMT protocol [39] were compared to a neutral memory condition among survivors of trauma. In the first study, authors found that participants who repeatedly narrated the content of positive autobiographical memories reported decreases in PTSD symptom severity and negative affect, as well as increases in positive affect across time compared to the control condition [38]. In the second study, authors found that survivors of trauma who repeatedly retrieved positive (and neutral) memories reported less PTSD and depression severity, fewer posttrauma cognitions, and improved affect [39]. Using an open-label pilot trial, the feasibility and effects of the 5-session PPMT were examined among 12 survivors of trauma [40,41]. The authors found that PPMT reduced PTSD symptoms, reduced negative affect, and improved regulation of positive affect, and there were good feasibility indicators for PPMT (eg, PPMT was acceptable).

Critically, no study has examined PPMT’s effects using a larger community sample, nor has there been any exploration of whether PPMT is associated with postintervention changes in how survivors of trauma react to events in daily life. We can hypothesize that PPMT may impact individuals’ daily-life affect and cognitions; these impacts represent hypothesized mechanisms through which PPMT may reduce PTSD symptom severity over time. Most studies examining PTSD intervention impacts use case-series designs, in which data are collected from a group of individuals pre- and postintervention and an aggregate assessment of symptomatology pre- and postintervention is conducted and compared. However, this approach has some noteworthy limitations. Affect, cognitions, and PTSD symptoms are dynamic and vary daily in response to trauma reminders and experiences [42-44]; thus, evaluating intervention effectiveness using 2 snapshot assessments of symptomatology is not sufficiently reliable or nuanced. Furthermore, case-series designs do not examine the daily-level mechanisms of change for an intervention. Given that PPMT may impact daily life affect, cognitions, and symptoms, it is crucial that the data enable an examination of these constructs at the daily level.

These limitations can be overcome by integrating case-series designs with a daily diary framework for the pre- and post-PPMT assessments. Daily diary studies are an intensive longitudinal data collection method in which participants provide daily reports of their experiences each day over a period of time. Compared to retrospective assessments, daily diary data are considered more ecologically valid as they are collected in an individual’s everyday life rather than in a laboratory, more accurate and robust, and less vulnerable to recall bias [45]. Thus, the proposed study outlines information on the protocol of an ongoing study that combines case-series design and daily diary approaches to provide novel insights into PPMT’s effects on daily-level cognitive and affective experiences. This approach can be conceptualized as a special subtype of case-series design that enables an examination of within-person changes at the daily level.

Specifically, the proposed study aims to use daily diary data pre- and post-PPMT to examine changes in daily PTSD, daily affect, and daily cognitions. We hypothesize that at the daily level, participants will report (1) a lower count of endorsed daily PTSD symptoms pre- to post-PPMT, (2) increases in daily positive affect and decreases in daily negative affect pre- to post-PPMT, (3) increases in positive affect reactivity to daily positive events (a within-person index of linear relations between daily positive events and daily positive affect) pre- to post-PPMT, and (4) decreases in daily posttrauma cognitions pre- to post-PPMT. The aim of this study is to detail the proposed research protocol and our hypotheses regarding daily PTSD, daily negative affect, and daily cognitions pre- and post-PPMT. As a supplementary analysis, we will examine if participants report a greater count of retrieved specific positive memories pre- to post-PPMT.

## Methods

### Study Design

**Figure 1:**
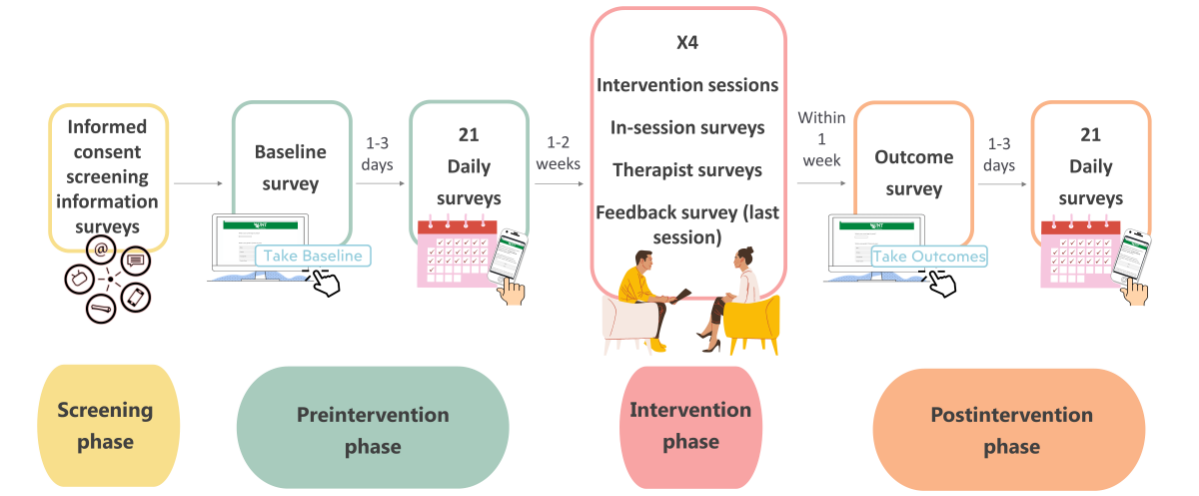
Study procedure: explaining the step-by-step process of the study.

The study involves four phases: (1) screening phase (eligibility survey), (2) preintervention phase (baseline survey and daily surveys), (3) intervention phase (PPMT and weekly surveys), and (4) postintervention phase (outcome survey and daily surveys). Figure 1 provides an illustration of the study procedure. All assessments are completed by participants using a computer or smartphone. Greene and colleagues [46] provide a detailed protocol for the questionnaires. Briefly summed up, following a web-based screening survey, eligible participants are asked to complete a preintervention phase baseline survey followed by 21 daily surveys in their natural environments. Then, they receive 4 PPMT sessions, completing 1 web-based survey per session and a feedback survey in the last session. After the conclusion of the PPMT intervention, participants are asked to complete a postintervention phase outcome survey and 21 daily surveys.

### Participants

Participants (target n=70) are currently being recruited from a metroplex in the southwest United States through social media postings, flyers at businesses and public places, and university announcements since March 2022. The inclusion criteria are: (1) being aged between 18 and 65 years; (2) endorsing a trauma with posttrauma symptoms assessed by the Primary Care PTSD Screen for DSM-5 [47]; (3) access to an electronic device (eg, a computer or a smartphone) with internet capabilities; (4) working knowledge of English; (5) no active suicidal plan, suicidal attempt, homicidal plan, or homicidal attempt (past 3 months including current); (6) being a current resident of the Dallas Fort Worth metroplex; (7) not currently in therapy with a mental health provider; (8) willingness and availability to participate in approximately 10 weeks of this study (including 4 therapy sessions); and (9) willingness to be video-recorded during sessions for quality control purposes.

### Procedure

#### Screening Phase

During this phase, interested participants complete an eligibility survey. First, they read the informed consent document (information about the study, eligibility criteria, compensation, benefits and risks to study participation, and steps to ensure data confidentiality) and provide electronic informed consent if they wish to participate. Next, they answer questions to determine eligibility. Eligible and consenting participants are automatically redirected to a separate survey, wherein they provide contact information for study purposes. Research personnel then contact eligible participants to provide more information on the study (eg, survey timelines and PPMT sessions). No compensation is provided for this study phase.

#### Preintervention Phase

Participants complete 1 baseline survey and 21 daily surveys as part of this phase. The baseline survey contains questions on demographics, trauma history, PTSD symptoms, and other psychological symptoms, as well as affect and cognitive processes (approximately 30-minute completion time). Eligible participants receive the baseline survey link by email at a date determined to be feasible based on contact with participants. Participants are given up to 48 hours to complete the baseline survey and are sent reminders if they do not complete the survey in a timely manner. Research personnel monitor survey responses for completion, response times to identify unfeasibly short times, accurate participant ID entry, therapy history to confirm they are not currently in therapy, and trauma history.

Participants who complete the baseline survey are asked to complete 21 daily surveys. The daily surveys include questions assessing daily PTSD symptoms, affect, cognitions, and events that occurred in the last 24 hours (approximately 3-5 minutes to complete each survey). The link to the first daily survey is emailed to participants within 1-3 days after completing the baseline survey. Participants receive daily surveys once a day at fixed intervals (at 7:00 PM each day) over a 21-day period, and they have until 11.59 PM to complete each daily survey. Participants are sent text reminders for survey completion to enhance compliance and are contacted if they miss any surveys.

#### Intervention Phase

PPMT is administered weekly as a 4-session protocol during the intervention phase. The sessions are scheduled within 1-2 weeks after completing the preintervention phase. In session 1, participants receive psychoeducation on PTSD symptoms, an overview of PPMT, and are assessed for psychological symptoms. Sessions 1-4 involve the detailed processing of a salient positive autobiographical memory to elicit “values, affect, strengths, and thoughts” related to that positive memory. Homework assignments include listening to an audio recording of that memory, completing a “values, affect, strengths, and thoughts” log, and engaging in a behavioral activity. In session 4, the therapist also reviews psychological symptoms and addresses termination. Following the completion of session 4, the participants complete a feedback survey on PPMT.

#### Postintervention Phase

Participants complete 1 outcome survey and 21 daily surveys as part of this phase. Within approximately 1 week after completing the intervention phase, participants complete an outcome survey. The procedural aspects of this phase mimic the preintervention phase. The outcome survey has questions similar to those of the preintervention phase baseline survey (without demographics and trauma history items and with different cue words for the measure examining the count of retrieved memories).

#### Dropout

Participants who do not complete the outcome survey after 2 days, consecutively miss 4 daily surveys, or miss more than 8 daily surveys (<60% of the daily surveys) in either the preintervention or postintervention phases are considered dropouts for this study, and they do not continue to receive survey links in order to avoid burdening participants with repeated requests to complete the surveys if they no longer wish to participate.

### Study’s Primary Measures

#### Overview of Primary Measures

In this section, we outline the measures that relate to the primary outcomes of this study. The primary outcomes of interest are daily positive affect levels, daily positive affect reactivity (within-person index of linear relations between daily positive events and daily positive affect [48]), daily negative affect levels, daily posttrauma cognitions, daily PTSD symptoms, and the number of retrieved specific positive memories as measured pre- and postintervention. Secondary measures (eg, difficulties in positive emotional regulation using the “Difficulties in Emotion Regulation Scale-Positive” and the severity of PTSD symptoms using the “PTSD Checklist for DSM-5”) are also administered to allow for the assessment of possible mediators and moderators of treatment effects. Table S1 in Multimedia Appendix 1 [40,47,49-58] provides detailed information on all study measures (including measures for supplemental analyses).

#### Preintervention Phase Baseline Survey

The number of retrieved specific-positive memories is measured by the Autobiographical Memory Test (AMT) [49,59]. The AMT uses a cued memory recall technique involving the presentation of individual cue words, followed by a prompt to recall a personally meaningful and specific memory of an event that took place within any 24-hour period. For this study, participants are shown cue words and asked to retrieve a personal and specific memory of the cue-word-related event within 60 seconds [59]. The instructions were adapted from previous autobiographical memory studies [49,60,61]. In the preintervention baseline survey, we included 5 cues drawn from previous studies: friendly, happy, honest, kind, and humorous [62-64]. We will follow coding guidelines to categorize AMT responses [65,66]. AMT responses will be coded as specific (event that occurred at a certain place within 24 hours), extended (event that lasted >1 day), or categorical (summary of repeated events). AMT responses will also be coded as positive or nonpositive following the Coding and Assessment System for Narratives of Trauma [67]. Lastly, AMT responses will be coded as semantic associate (no personal memory) and omission (did not retrieve the memory within 60 seconds or was unable to recall a memory). The AMT demonstrates good psychometrics [68].

#### Preintervention Phase Daily Surveys

Daily negative and positive events are measured by asking participants to rate their most positive and negative events in the last 24 hours from 0 (not at all unpleasant) to 3 (very unpleasant) [50].

Daily affect levels (ie, positive and negative) are assessed by rating the extent of 4 positive (excited, cheerful, satisfied, and relaxed) and 6 negative (stressed, irritated, anxious, sad, hopeless, and insecure) emotions in the last 24 hours. These emotions were used in a previous daily diary study based on a theoretical circumflex of emotions. The study showed excellent between-person reliability and good within-person reliability [69]. In this study, responses are rated on a 5-point Likert scale ranging from 1 (very slightly or not at all) to 5 (extremely).

Daily posttrauma cognitions are measured by the Brief Version of the Posttraumatic Cognitions Inventory [51], which is a 9-item self-report measure assessing posttrauma cognitions. Responses are provided on a 7-point Likert scale ranging from 1 (totally disagree) to 7 (totally agree), with the time frame modified to “in the last 24 hours.”

Daily PTSD symptom severity is assessed using the Primary Care PTSD Screen for DSM-5 [47], which is a 5-item self-report measure with dichotomous “yes” or “no” items; the time frame is modified to “in the last 24 hours.” We will use the count of endorsed PTSD symptoms as a measure of daily PTSD symptom severity.

#### Postintervention Phase Outcome Survey

Similar to the preintervention phase baseline survey, the AMT is administered to examine the number of retrieved specific positive memories. Different cue words are used: peaceful, loyal, helpful, safe, and love [62-64].

#### Postintervention Phase Daily Surveys

The procedural aspects and measures mimic the preintervention phase daily surveys.

### Participant Safety and Psychological Distress

There are minimal foreseeable risks associated with this study. Few participants may experience an increase in PTSD severity [70] or suicidal ideation [71], attributed to the sensitive nature of questions targeting trauma reactions and to PPMT itself. To address any such concerns, the study co-principal investigator (co-PI; a licensed clinical psychologist) is training all research personnel on considerations in administering assessments to survivors of trauma, providing clinical training and weekly supervision to study therapists, and training study therapists in anxiety-reduction techniques (eg, guided imagery). Furthermore, study participants receive information on mental health services.

To address any risk factors for self-harm, we are closely monitoring participants during PPMT to assess for any reported suicidal ideation, plan, or attempt (eg, administration of a depression measure every session). Furthermore, the study co-PI is training study therapists to conduct in-depth suicide risk assessments. In the event of suicidal ideation, plan, or attempt reported by participants in session, study therapists conduct a suicide risk assessment and consult with the supervising co-PI to determine if emergency services need to be contacted to ensure participant safety. Also, the participant or therapist may discontinue the study at any time should symptoms worsen or if the participant simply desires to withdraw. Lastly, we are providing information on community mental health centers offering 24-hour access to services and emphasizing contacting 911 or 988 in times of imminent risk.

### Treatment Nonresponse or Relapse

Any treatment for posttrauma mental health is associated with some chance of failure to respond or relapse [72]. We are implementing the following procedures to address any such potential concerns. If a participant shows substantial increases in PTSD or depression severity or reports risk factors for self-harm during intervention sessions, we are providing mental health referrals immediately for alternate treatment options, including a 24-hour access local mental health center. Furthermore, research personnel are contacting participants when they miss appointments to check on their health status.

### Intervention Training

#### Therapist Training

The study co-PI trained doctoral students (ie, study therapists) in PPMT. This training included a review of PPMT’s theoretical underpinnings, manuals, and fidelity checklists, as well as practice in PPMT administration. Furthermore, study therapists are required to follow detailed session protocols during each PPMT session. Research assistants were trained in PPMT fidelity ratings; ≥0.81 kappa coefficient and ≥0.8% percent agreement will be considered acceptable interrater reliability (IRR) [73].

#### Treatment Delivery

All sessions are being video recorded. The co-PI has reviewed all recorded PPMT sessions for 1 participant for each study therapist; she will continue to review 20%-50% of the video-recorded sessions as needed [74]. The co-PI is providing weekly group supervision to study therapists that involves case discussions and feedback.

#### Fidelity Ratings Across Raters

The authors have created fidelity checklists that include a list of proscribed PPMT components to be recorded as occurring or not occurring. Using these fidelity checklists, 2 trained evaluators will independently code video-recorded sessions for 18-20 participants. These data will be used to compute IRR estimates. If acceptable IRR estimates are not achieved, trained evaluators will code an additional 20% of sessions. Once acceptable IRR estimates are achieved, the evaluators will solely code the remainder of the treatment sessions for fidelity.

#### Adherence to PPMT Components

We will compute percentage adherence across sessions for each of the trained study therapists, with the recommended 80%-100% benchmark indicating high fidelity [74].

### Data Analysis

#### Power Analysis

We conducted an a priori power analysis using the *EMAtools R* (Kleiman) package [75] for power curves for multilevel studies. The power analysis was based on two 3-week assessment bursts with 1 questionnaire per day and an estimated intraclass correlation coefficient of 0.36 based on a previous study on daily-level emotions, cognitions, and PTSD [76]. Analysis showed that 70 participants and up to 25% missing data would be sufficient to detect a medium effect size (*d*=0.5) with 80% power.

#### Analytical Plan

A paired sample *t* test will be used to examine changes in the count of retrieved specific-positive autobiographical memories pre- versus post-PPMT (comparing the preintervention baseline and postintervention outcome surveys). To examine changes in daily affect, daily cognitions, and daily PTSD symptoms, we will use the daily diary reports pre- and postintervention and conduct multilevel random intercepts and slopes linear regression models for each outcome variable using *MPlus 8.*3 (Muthén and Muthén), *nlme* (Pinheiro et al), and *lme R* (Bates et al) packages, comparing the models pre- and post-PPMT with and without demographic covariates (eg, gender, age, and education). We will also conduct exploratory analyses examining additional variables included in the study as predictors or moderators of post-PPMT outcomes (eg, count of trauma types previously experienced, PTSD severity at baseline, and difficulties in positive emotional regulation).

#### Missing Data

At the survey level, the web-based questionnaire has been set up with a prompt if questions have been skipped, with the option to continue the survey without completing a particular item or to go back and complete the skipped question. We anticipate a little missing data within the submitted surveys. We will treat surveys that have been submitted with missing data as complete for the purposes of determining dropout and participation compensation. An analysis will be conducted to investigate the pattern of missingness. If data are missing at random or missing completely at random, they will be handled by listwise deletion and models fit by maximum likelihood. If data are not missing at random, then missing data will be imputed using the MICE (van Buuren et al) package in R [77] and fit by maximum likelihood.

### Ethical Considerations

The institutional review boards at the University of North Texas (#21-420) and the University of Haifa (#480/21) have approved this study. During the screening phase, interested participants read the informed consent document (information about the study, eligibility criteria, compensation, benefits and risks to study participation, and steps to ensure data confidentiality) and provide electronic informed consent if they wish to participate. Participants are then contacted by research personnel to re-explain any study procedures and obtain or confirm identifying information.

In terms of compensation, participants receive US $1.50 for each completed daily survey and US $10 each for completing each of the baseline and outcome surveys. Participants receive US $10 for completing each of the 4 PPMT session surveys and US $12 toward transportation costs cumulatively for all 4 (attended) intervention sessions. In order to incentivize participants to provide as much data as possible, participants who complete 36 surveys without any missing data receive an additional US $15. The total potential compensation for participation is US $150.

Participants provide personally identifiable information (eg, name and contact information), which is only used for scheduling purposes, study-related communications, and to connect data longitudinally. Each participant receives a unique and randomly generated ID number, which is used on all web-based surveys for this study. At no point is any personally identifiable information linked to participant data. Furthermore, deidentified data will be analyzed for the scientific dissemination of study findings.

## Results

Year 1 of the study (October 2021-September 2022) was primarily devoted to recruiting and training research personnel, obtaining ethics approvals, and preparing to launch the study. Year 2 of the study (October 2022-September 2023) has been focused on participant recruitment and data collection. During Year 3 of the study (October 2023-September 2024), we will complete data collection from our targeted sample and start the data cleaning and analysis process. During Year 4 of the study (October 2024-October 2025), we will complete the data analyses and prepare planned scientific outputs (eg, publications and presentations).

Data collection was initiated in March 2022. As of November 28, 2023, a total of 515 participants had consented to the study in the screening phase. Of those, 258 (50.1%) participants were eligible, 92 (35.7%) of which gave their consent by phone and attempted the first daily survey of the study. Of the 92 participants who completed the first daily survey, a total of 58 (63%) participants completed all intervention sessions and ≥13 daily surveys (≥60% of the daily surveys), and 28 (30.4%) participants dropped out in various stages of the study, mostly before the PPMT intervention. As of November 29, 2023, a total of 6 (6.5%) participants are currently participating in various phases of the study. Data collection is expected to end by June 2024. No analyses will be conducted until data collection has been completed.

## Discussion

This study aims to examine the daily-level impacts of PPMT, a promising adjunct or alternative to traditional PTSD treatments, in a trauma-exposed, nonclinical community sample. This study combines a case-series design and a daily diary design to examine potential mechanisms of change in PTSD symptoms by assessing daily affect, daily cognitions, and daily PTSD symptoms before and after the PPMT intervention. This approach enables a more nuanced and ecologically valid exploration of changes as compared with retrospective aggregate assessments. We outline the research protocol for this study, including the hypotheses and the proposed analyses. When data collection has been completed (estimated date: June 2024), we will test our hypotheses that, at the daily level, in comparison to their own pre-PPMT levels, following the PPMT intervention, participants will report (1) a lower count of endorsed daily PTSD symptoms, (2) increases in daily positive affect and decreases in daily negative affect, (3) increases in positive affect reactivity to daily positive events, and (4) decreases in daily posttrauma cognitions.

The findings of this proposed study could have significant implications. Results could clarify whether deficits in positive autobiographical memory processes (eg, retrieval and encoding) may also characterize PTSD alongside deficits in traumatic memory processes [7]. If the study’s hypotheses are confirmed, PPMT could be an additional therapeutic tool for clinicians to help clients with posttraumatic distress. Unlike other trauma interventions, PPMT exclusively targets positive autobiographical memories in treatment while redirecting attention away from negative content embedded in the positive memories. By uniquely combining positive and symptom-focused techniques and theories, PPMT aims to increase positive elements (eg, values, affect, and thoughts) while simultaneously decreasing PTSD severity [17].

There are some limitations to this study that should be considered. The study uses a self-report approach, which, although it reduces recall bias, is still subject to potential difficulties in the recall of experiences over the course of each day. Relatedly, while it reduces participant burden, the PC-PTSD measure is a PTSD symptom screener and usually is coupled with a comprehensive structured diagnostic interview for PTSD or a self-report measure assessing all 20 PTSD symptoms (which we do not do in this study due to the daily-level methodology and associated participant and time burdens). Furthermore, we are not gathering information on trauma characteristics such as the ages at which the trauma was experienced, the frequency or chronicity of each experienced trauma, or the time since the trauma has elapsed. Such information can impact posttrauma distress [78-80] and may moderate the impacts of PPMT in this study; hence, it should be empirically investigated in future research. In addition, while the research design of one assessment per day enables examination of changes in daily symptoms, affect, and cognition before and after the intervention, it does not have a sampling frequency nor sufficient power to examine fine-grained dynamic interactions between symptoms, affect, and cognition using even more complex modeling techniques. Lastly, our eligibility criterion permits individuals endorsing even 1 PTSD symptom at a clinical level to be included in the study. While such an approach accounts for impairment among individuals endorsing sub-threshold PTSD [81], it may also make it statistically difficult to detect any changes in PTSD symptoms (ie, the floor effect).

In conclusion, this study will contribute to the development of more personalized and alternative PTSD interventions for survivors of trauma who drop out or do not benefit from existing PTSD treatments. Further studies could examine PPMT as an ecological momentary intervention, wherein individuals receive daily reminders and instructions for engaging in therapy-relevant behaviors (eg, processing of positive memories). Such interventions can particularly benefit communities that do not have easy access to mental health services and are underserved in that regard [82]. Finally, this study will give insight into the mechanisms of change in the PPMT intervention through elucidating daily-level changes in affect, cognition, symptoms, and event reactivity.

## Appendix

Multimedia Appendix 1 Supplemental table 1. Timeline information on measures.

## Abbreviations

AMT: Autobiographical Memory Test
co-PI: coprincipal investigator
IRR: interrater reliability
PPMT: Processing of Positive Memories Technique
PTSD: posttraumatic stress disorder

## Data Availability

The data sets that will be generated through this study will not be publicly available due to the sensitive nature of the posttrauma daily-level data, however the corresponding author will make deidentified data available on reasonable request.
