# Supplementary material for: The Effects of the Processing of Positive Memories Technique on Posttrauma Affect and Cognitions Among Survivors of Trauma: Protocol for a Daily Diary Study
Source: JMIR Res Protoc. 2024 Jan 12;13:e51838. doi: 10.2196/51838 (PMC10818235; doi:10.2196/51838)
Supplement: Multimedia Appendix 1 [file resprot_v13i1e51838_app1.docx]

**Table S1.** Timeline information on measures.

| Measures | Screening Phase | Pre-Intervention Phase  Baseline Survey | Pre-Intervention Phase  Daily Survey | Intervention Phase | Post-Intervention Phase Outcome Survey | Post-Intervention Phase Daily Survey |
| --- | --- | --- | --- | --- | --- | --- |
| Primary Care PTSD Screen for DSM-5 (PC-PTSD-5) [47] | ▪ |  |  |  |  |  |
| Demographic Questionnaire |  | ▪ |  |  |  |  |
| Life Events Checklist for DSM-5 (LEC-5) [52] |  | ▪ |  |  |  |  |
| PTSD Checklist for DSM-5 (PCL-5) [53] |  | ▪ |  | ▪ | ▪ |  |
| Brief Version of the Posttraumatic Cognitions Inventory (PTCI-9) [51] |  | ▪ |  | ▪ | ▪ |  |
| Patient Health Questionnaire–9 (PHQ-9) [54] |  | ▪ |  | ▪ | ▪ |  |
| Positive and Negative Affect Schedule (PANAS) [55] |  | ▪ |  | ▪ | ▪ |  |
| Difficulties in Emotion Regulation Scale – Positive (DERS-P) [56] |  | ▪ |  | ▪ | ▪ |  |
| Autobiographical Memory Test (AMT) [49] |  | ▪ |  |  | ▪ |  |
| Attitude towards Trauma-Focused Intervention and Memory Processing [40] |  | ▪ |  |  | ▪ |  |
| Memory Experiences Questionnaire–Short Form (MEQ-SF) [57] |  |  |  | ▪ |  |  |
| Daily negative and positive events [50] |  |  | ▪ |  |  | ▪ |
| Feedback on PPMT [52] |  |  |  | ▪ |  |  |
| Daily Positive and Negative Affect [55] |  |  | ▪ |  |  | ▪ |
| Daily Primary Care PTSD Screen for DSM-5 (Daily-PC-PTSD-5) [47] |  |  | ▪ |  |  | ▪ |
| Daily Posttraumatic Cognitions Inventory (Daily-PTCI-9) [51] |  |  | ▪ |  |  | ▪ |

*Notes*. PC-PTSD-5 is a 6-item self-report measure of lifetime traumatic experiences and five past-month PTSD symptoms; Demographic questions included the following: age, gender, ethnicity, racial background, educational level, income level, employment status, relationship status, and therapy status. LEC-5 is a 17-item self-report measure evaluating lifetime traumatic events; PCL-5 is a 20-item self-report measure of PTSD symptom severity cued to the past month; PTCI-9 is a 9-item self-report measure assessing post-trauma cognitions; PHQ-9 is a 9-item self-report measure of depression symptoms; PANAS is a is a 20-item self-report measure consisting of two 10-item subscales, one assessing positive affect and the other assessing negative affect; DERS-P is a 13-item self-report measure of positive emotion dysregulation; AMT assesses the number of retrieved specific positive memories by using five different cue words (Pre-Intervention Baseline survey: friendly, happy, honest, kind, humorous; Post-Intervention Outcome survey: peaceful, loyal, helpful, safe, and love) followed by a prompt to recall a personally meaningful and specific memory of an event that took place within any 24-hour period; Three questions are used to examine attitudes towards trauma-focused interventions and memory processing (created by research group); MEQ-SF is a 31-item self-report measure examining 10 phenomenological characteristics of a retrieved positive memory; Daily negative and positive events are measured by two items rating the extent of the most negative and most positive event of the day; The "Feedback on PPMT” survey includes quantitative questions and open-ended questions on PPMT’s feasibility, format, and content (created by research group); Daily Positive and Negative Affect are assessed by rating the extent of four positive (excited, cheerful, satisfied, relaxed) and six negative (stressed, irritated, anxious, sad, hopeless, insecure) emotions.

All daily surveys were tailored to the past 24 hours.
